# O3.6. DEFICITS IN CONTEXT-DEPENDENT ADAPTIVE CODING IN EARLY PSYCHOSIS AND HEALTHY INDIVIDUALS WITH SCHIZOTYPAL PERSONALITY TRAITS

**DOI:** 10.1093/schbul/sby015.204

**Published:** 2018-04-01

**Authors:** Matthias Kirschner, Amelie Haugg, Andrei Manoliu, Erich Seifritz, Philippe N Tobler, Stefan Kaiser

**Affiliations:** 1 University Hospital of Psychiatry, University of Zurich; 2 University of Zurich; 3 University Hospitals, Switzerl

## Abstract

**Background:**

Adaptive coding of reward values is a fundamental principle of brain functioning to efficiently represent a theoretically infinite range of rewards in the natural environment with the limited coding range of reward-processing neural machinery. Patients with schizophrenia show impaired neural adaptation to the current reward context. However, it is unknown if and how generally this impairment extends across the psychosis spectrum.

**Methods:**

We studied 27 patients with first-episode psychosis, 26 individuals with schizotypal personality traits and 25 healthy controls using functional magnetic resonance imaging in combination with a variant of the monetary incentive delay task. We assessed adaptive reward coding in two reward conditions with different reward ranges.

**Results:**

Compared to healthy controls, patients with first-episode psychosis and individuals with schizotypal personality traits showed less efficient neural adaptation to the current reward context in the caudate. The two groups therefore showed a similar deficit in reward representation as patients with schizophrenia. In addition, we find impaired adaptive coding of reward in the caudate and putamen to be associated with total symptom severity across the psychosis continuum.

**Discussion:**

Deficits in adaptive coding were prominent across the psychosis continuum and even detectable in unmedicated healthy individuals with schizotypal personality traits. In addition, the association between total symptom severity and impaired adaptive coding in the right caudate and putamen suggests a dimensional mechanism underlying imprecise neural adaptation. Our findings support the idea that impaired adaptive coding may be a general information-processing deficit across the psychosis spectrum and not limited to schizophrenia.

